# An exploratory pre-post test evaluation of an online family cooking intervention: Up for Cooking

**DOI:** 10.1017/jns.2025.10034

**Published:** 2025-09-08

**Authors:** Lisa S.E. Harms, Jessica S. Gubbels, Patricia van Assema, Sanne M.P.L. Gerards, Kathelijne M.H.H. Bessems

**Affiliations:** Department of Health Promotion, Research Institute of Nutrition and Translational Research in Metabolism (NUTRIM), Maastricht University, PO Box 616, 6200 MD Maastricht, The Netherlands

**Keywords:** Cooking skills, Food literacy, Internet-based intervention, Parents, SES, socio-economic status, FL, food literacy, CL, course leader, SD, standard deviation

## Abstract

This pilot study evaluated the effect of an online cooking intervention: Up for Cooking. Seventy-three Dutch families participated in four 1.5-hour sessions, before which they received ingredients and intervention materials. Parental questionnaires (pre-post) assessed food literacy skills (planning, selecting and making a healthy meal), knowledge and self-efficacy towards cooking and healthy eating (quantitative). Interviews assessed whether families changed their cooking behaviour at home (qualitative). A Wilcoxon Signed Rank test and inductive thematic coding were used. Thirty-nine parents completed questionnaires and eleven parents participated in interviews. Scores on food literacy items related to selecting and making a healthy meal improved significantly post-intervention. Parents’ knowledge of healthy eating and self-efficacy in cooking with their children also improved significantly. Interviews revealed an increased involvement of children in meal preparation and positive changes in family cooking behaviour. This online cooking intervention is a promising nutrition intervention, but implementation and long-term changes need further exploration.

Unhealthy dietary behaviours are risk factors for the development of chronic diseases and high mortality rates worldwide^(1)^. In the Netherlands, adherence to the dietary guidelines is low^(2)^. Food literacy (FL) has emerged as an important modifiable determinant of dietary behaviour^(3)^. FL encompasses the knowledge, skills and behaviours necessary for a healthy diet^(4)^, and includes four key domains: *Planning food intake* (e.g., making a grocery list), *Selecting food* (e.g., reading food labels), *Preparing food* (e.g., basic hygiene principles), and *Eating food (*e.g., social aspect of eating)^(4)^. Thus, FL captures the complexity of healthy eating: from beliefs and knowledge about healthy eating, to the skills and sub-behaviours needed to consume a healthy meal. Previous studies have shown a positive association between FL and dietary intake^(5)^. For example, positive associations have been found between higher levels of FL and fruit, vegetable and fish consumption in Dutch adults^(6)^ and with healthy dietary intake in general^(7)^. This makes FL a promising target for nutrition interventions^(5,8)^.

Cooking interventions have been successful in reaching and engaging adults and families^(9–12)^. They can involve children in meal preparation^(13)^, which can lead to improved eating enjoyment^(14)^ and family meal frequency^(15)^. Furthermore, beneficial effects have been found on FL^(16)^ and on cooking confidence, knowledge, skills, diet quality and health status^(17)^. To our knowledge, there is limited research on FL cooking interventions in the Dutch setting. This study explores the effects of a food literacy cooking intervention: ‘Up for Cooking (UfC) online’. It examines the associations between UfC online and changes in parental FL, knowledge and self-efficacy, and whether families changed their cooking behaviour at home.

## Methods

### Study design and setting

This study was conducted in the Netherlands. A mixed-methods approach combined parental pre-questionnaires (one week before participation) and post-questionnaires (one-two weeks after participation) with interview data (after participation). The Faculty of Health, Medicine and Life Science Research Ethics Committee of Maastricht University has approved this study (FHML-REC/2020/067). A more detailed description of the intervention and measurements can be found in the Open Science Framework Repository at https://osf.io/4mdsh/.

### Intervention

UfC online consisted of four 1.5-hour online cooking sessions for five to eight families. A trained course leader (CL) guided parent-child pairs through a recipe, while offering practical tips and preparing the meal herself. Prior to each session, participants received a grocery bag with the ingredients, recipes, homework assignments and access to the videoconferencing software (Zoom Business). Recipes differentiated tasks for parents and children. Homework assignments used easy-to-read language, pictures and step-by-step tasks.

### Participants

Primary schools recruited families through the children (generally aged 8–12). Recruitment was aimed at one parent and one child per family (i.e., parent-child pairs), but other family members could be present and participate. The research team invited participating parents to complete the questionnaires and, if they attended at least 3 out of 4 cooking sessions, for an interview.

### Measurements

Questionnaires assessed FL, socio-cognitive determinants, self-reported impact and demographic characteristics. FL included 14 items derived from a validated FL behaviour checklist, covering the FL domains *planning*, *selecting* and *making* ^(18)^. Items asked how often participants performed the listed behaviours (e.g., trying a new recipe) in the past month, ranging from [1] ‘never’ to [4] ‘always’. Self-reported knowledge about healthy eating was assessed by a single item, and four items addressed self-efficacy, both ranging from [1] ‘No, definitely not’, to [5] ‘Yes, definitely’. Lastly, demographic measures included the respondent’s age, country of birth, family status, highest level of education completed, postal code, and the number and age of children in the household. Semi-structured interviews assessed self-reported impact on family cooking behaviours at home.

### Data processing and analyses

Single FL items were assigned to one of the FL domains based on Begley (2018)^(18)^. Scale reliability was assessed using Cronbach’s alpha, using a cut-off of α ≥ 0.5, as acceptable given the small sample size^(19)^. Items were deleted from the scale if this improved Cronbach’s alpha to above the cut-off. Sum scores were calculated and divided by the number of scale items. Wilcoxon signed rank tests were used to compare pre- and post-questionnaire scores.

The qualitative interviews were transcribed verbatim, anonymised, and coded using inductive thematic analysis in NVivo 12 (QSR International, Doncaster, Victoria, Australia). The first author analysed all interviews. A second researcher (LV) independently coded 18% (two out of eleven) of the interviews, for which intercoder reliability was found to be 0.98.

## Results

### Participants, response rate and demographics

Of the 73 participating families, 54 (74%) completed the pre-questionnaire and 39 (53%) completed both pre- and post-questionnaires. Dropout analyses revealed significant differences in parental age (F(1, 52)= 5.35, p=0.025) and neighbourhood SES score (F(1, 49)=4.71, p=0.035). Parents who completed both questionnaires were older (41.6 compared to 37.6 years) and came from neighbourhoods with higher SES scores (–0.8 compared to –1.8) than parents who completed only the pre-questionnaire. Eleven parents (15%) participated in semi-structured interviews, of whom two completed only the pre-questionnaire.

The mean age of the respondents was 41.6 (SD=6.2) years. The neighbourhood SES score ranged from –3.6 to 0.9, with a negative score indicating a lower SES as the national average is zero. The majority of respondents were born in the Netherlands (94.7%) and had an average of 2.4 (SD=1.0) children in their household.

### Food literacy

Changes in parental FL are shown in Table 1, described in more detail below and supplemented by parent quotes from the interviews in Table 2.

**Table 1.** Parents’ food literacy, knowledge and self-efficacy before and after participation in Up for Cooking online (n=39)

Category and single item	Cronbach alpha^a^	Mean scores (SD)		Pre-post change		Wilcoxon	
		Pre	Post	Mean^b^	%	Z^c^	p
*Food literacy*							
*How often in the last month did you…* ^*d*^							
Plan (total scale score)	0.533	3.02 (0.49)	3.10 (0.51)	0.08	2.72	–1.22	0.221
Plan meals in advance		3.21 (0.66)	3.16 (0.80)	–0.04	–1.34	–0.30	0.763
Make a shopping list		3.05 (1.00)	3.14 (1.00)	0.08	2.75	–1.13	0.257
Plan meals to include all food groups		3.03 (0.71)	3.19 (0.71)	0.17	5.58	–1.11	0.268
Thought about healthy food choices		2.79 (0.70)	2.92 (0.60)	0.12	4.44	–0.73	0.467
Select (total scale score)	0.708	1.74 (0.69)	2.05 (0.78)	0.31	17.81	–2.38	**0.017**
Use of a nutrition information panel		1.77 (0.78)	2.05 (0.82)	0.28	16.10	–2.30	**0.022**
Use other parts of food label		1.74 (0.79)	2.05 (0.85)	0.32	18.26	–1.96	0.051
Make (total scale score)^e^	0.171						
Cook with healthy ingredients		3.21 (0.57)	3.41 (0.55)	0.20	6.25	–2.14	**0.033**
Try a new recipe		2.08 (0.48)	2.16 (0.37)	0.09	4.10	–1.00	0.317
Make recipes healthier		1.74 (0.76)	2.14 (0.69)	0.41	23.37	–3.13	**0.002**
*Knowledge*							
I know what to do in terms of healthy eating^f^		4.03 (0.84)	4.46 (0.61)	0.43	10.77	–3.13	**0.002**
*Self-efficacy*							
*Are you able to …* ^*f*^							
Cook healthy meals for your family?		4.46 (0.72)	4.57 (0.60)	0.11	2.38	–1.16	0.248
Eat healthy with your family?		4.38 (0.75)	4.49 (0.65)	0.10	2.32	–1.39	0.166
Cook with your child?		4.44 (0.75)	4.84 (0.37)	0.40	9.06	–3.12	**0.002**

Abbreviations: SD: standard deviation.

^a^Calculated over post-scores. ^b^Calculated as post-pre mean scores due to missing data, and rounded.^c^Based on negative ranks, see Supplementary Table. ^d^Scored from [1] ‘Never’ to [4] ‘Always’. ^e^Sum scale not calculated due to unreliable scale. ^f^Scored from [1] ‘No definitely not’ to [5] ‘Yes, definitely’. Significance levels in Wilcoxon Signed-rank test.

**Table 2.** Impact at home from quotes by parents (n=11)

Construct	Theme	Example quote
Food literacy		
Planning	Daily routines	‘I am quite structured. I always have to have a weekly schedule and menu’
Select	Homework assignments as cue to action	‘We immediately put the sticker [indicating how to store 12 commonly used products] on our fridge. Since then I have also bought a basket, tomatoes and cucumber are no longer stored in the fridge’
	In-depth information	‘[CL should] also explain some differences, why it is better to select wholemeal pasta than ‘white’ pasta’
Make	Healthier food swaps	‘I have read about healthier food swaps; (…) you really didn’t taste the difference. I think it is the idea you have in mind, of what your meal should look like’
Knowledge, self-efficacy and family cooking behaviours		
Knowledge	Eye-opener	‘Oh, they eat that! (…) wait a minute, maybe we can reintroduce that’
Self-efficacy	Parental expectations	‘Maybe, as a parent, you want to protect them so you didn’t allow certain things before. But they can do more than you think’
	Cooking skills	‘Children often say ‘I don’t eat Brussel sprouts’, but you can prepare Brussel sprouts in many other ways to make them tastier. (…) It is difficult, I would have liked to get some more tips on this topic’
Family cooking behaviours	Daily routines	‘I have a lot of phone calls, meetings, and consultations during the week (…) so cooking together doesn’t happen. That’s what I liked about having a fixed time, just like other hobbies, you make time for it’
	Children initiating changes	‘She said ‘mama, if you’re going to make something, I want to come and help, we’ll do it together’ ‘
	Ripple effect	‘We have another son, he sees it too. He just asked me ‘Mom, I’ll help you in a minute, what are we going to make?’ That is just nice’

No significant changes in *planning* were found (Table 1). Planning meals in advance and making a grocery list seems to already be part of daily routines. Significant increases were found in *selecting*. More specifically, interviews indicated that the homework assignment on food labels was appreciated (n=7), with explicit mention of the ‘back’ of food packaging (n=3), expiration dates (n=2), and proper refrigeration of food products (n=4). Although parents reported that they reviewed the homework assignment with their children, one parent preferred more in-depth information (Table 2). Two single items concerning *making* showed significant improvements. Interviews often referred to the different cutting techniques and healthy food swaps (i.e., wholemeal products). None of the parents reported trying new recipes, but instead applied the lessons learned instead.

### Knowledge, self-efficacy and family cooking behaviours

Knowledge of healthy eating and self-efficacy in cooking with their child improved statistically significant (Table 1). Parents considered it a learning experience to cook together with their child, reinforced by the separate parent and child cooking tasks in the recipes and instructions. Others missed information such as tips on different preparation methods (Table 2).

The fixed date and time helped parents to prioritise cooking with their child and to overcome barriers. Although parents often saw cooking or baking together as a weekend activity when they had more time, children initiated changes by preparing recipes or wanting to help parents. Overall, children initiating change was a recurring theme in the interviews, whether by reminding their parents of tips from the CL, UfC materials, food labels on products, or asking to be in charge of plating the food. Several parents also reported a ripple effect to others, including grandparents joining for dinner, classmates and friends coming to help during the sessions, or other household members curious about the next recipe (Table 2).

## Discussion

This study explored whether UfC online was associated with changes in parental FL, knowledge and self-efficacy, and whether families changed their cooking behaviour at home. Mixed results were found and direct effects were all relatively small. Self-reported impact at home showed increased involvement of children and other family members in cooking.

Despite the limited session duration, UfC online resulted in small positive changes in using nutrition information panels (i.e., *selecting*), making recipes healthier (i.e., *making*) and knowledge about healthy eating. This is comparable with findings from other cooking interventions^(20)^ and FL interventions^(21–23)^. No changes were found in *planning* healthy meals and parental self-efficacy towards cooking and eating healthy meals, potentially due to high pre-questionnaire scores with limited room for improvement. By providing families with a grocery bag containing the necessary ingredients, the practice of planning meals was eliminated. Other strategies, such as action planning, may be needed to achieve observable changes^(21,23)^.

UfC online supported parents in cooking with their child and promoted children’s involvement in cooking at home. This is important as it has been shown to have a positive effect on children’s dietary intake^(14,24)^. Cooking in their own kitchen, using their own equipment, and the child-friendly explanations may have contributed to this. It may have reduced parental concerns about safety issues (e.g., using a knife) or lack of control in the kitchen (e.g., children are a distraction, kitchen is a mess)^(25,26)^. Indeed, children themselves emphasise the need for age-appropriate tasks^(26)^. Parents and children experienced UfC online as valuable family time, which has also been observed in other cooking interventions^(27)^, overcoming competing schedules and affordability^(26,28,29)^. The ripple effect on other family members and friends is consistent with findings from other interventions^(30)^.

Strengths of the present study include the mixed-methods approach, the FL items derived from a validated questionnaire^(18)^, and the fact that data-saturation was reached in the interviews. Limitations include the lack of a control group, prohibiting the interpretation of intervention effectiveness as changes observed may have been caused by something other than the intervention. Collecting the post-questionnaire directly after the intervention may also have influenced the observed changes, given that participants were asked to reflect on the previous month. Finally, although the study achieved the required sample size to detect differences in FL, the small number of participants meant that adjustments for characteristics could not be made. The findings should be interpreted with caution. The effectiveness of UfC online should be further investigated with a longer follow-up, in a larger sample, including a control group.

Despite the increased interest in online (cooking) interventions since COVID-19, there are only a limited number of studies evaluating the impact of such interventions^(23,31,32)^. This evaluation, as well as another online cooking intervention that reported a waiting list of families willing to participate^(30)^, confirms that virtual live interventions may be a new step in nutrition interventions.

In conclusion, this study provides preliminary evidence that participation in UfC online was associated with short-term changes over time in parents’ FL, knowledge about healthy eating, and self-efficacy for cooking with their child. The intervention also seemed to lead (indirectly or directly) to changes in family cooking behaviour at home, such as involving other family members in meal preparation.

## Supporting information

Harms et al. supplementary material Harms et al. supplementary material

## Data Availability

Datasets used and/or analysed during the current study are available from the corresponding author on reasonable request.

## Supplementary material

The supplementary material for this article can be found at https://doi.org/10.1017/jns.2025.10034
