# Supplementary material for: An exploratory pre-post test evaluation of an online family cooking intervention: Up for Cooking
Source: J Nutr Sci. 2025 Sep 8;14:e63. doi: 10.1017/jns.2025.10034 (PMC12418272; doi:10.1017/jns.2025.10034)
Supplement: Harms et al. supplementary material [file S2048679025100347sup001.docx]

**Supplementary Table**. Wilcoxon Signed-rank test, exploratory evaluation before and after participation in Up for Cooking online (n=39)

| **Category and single item** | **Median** | | | **Positive ranks** | | | **Negative ranks** | | |  | |
| --- | --- | --- | --- | --- | --- | --- | --- | --- | --- | --- | --- |
|  | Pre | Post | Difference | N | Mean | Sum | N | Mean | Sum | Z ^1^ | *p* |
| ***Food literacy*** *How often in the last month did you… ^2^* |  |  |  |  |  |  |  |  |  |  |  |
| **Plan (total scale score)** | 3.00 | 3.00 | .00 | 16 | 13.88 | 222.00 | 10 | 12.90 | 129.00 | -1.22 | 0.221 |
| Plan meals in advance | 3.00 | 3.00 | .00 | 5 | 6.00 | 30.00 | 6 | 6.00 | 36.00 | -0.30 | 0.763 |
| Make a shopping list | 3.00 | 3.00 | .00 | 5 | 4.00 | 20.00 | 2 | 4.00 | 8.00 | -1.13 | 0.257 |
| Plan meals to include all food groups | 3.00 | 3.00 | .00 | 9 | 8.67 | 78.00 | 6 | 7.00 | 42.00 | -1.11 | 0.268 |
| Thought about healthy food choices | 3.00 | 3.00 | .00 | 8 | 7.88 | 63.00 | 6 | 7.00 | 42.00 | -0.73 | 0.467 |
| **Select (total scale score)** | 1.50 | 2.00 | .50 | 19 | 15.00 | 285.00 | 8 | 11.63 | 93.00 | -2.38 | **0.017** |
| Use of a nutrition information panel | 2.00 | 2.00 | .00 | 13 | 9.31 | 121.00 | 4 | 8.00 | 32.00 | -2.30 | **0.022** |
| Use other parts of food label | 2.00 | 2.00 | .00 | 16 | 11.38 | 182.00 | 6 | 11.83 | 71.00 | -1.96 | 0.051 |
| **Make (total scale score) ^3^** |  |  |  |  |  |  |  |  |  |  |  |
| Cook with healthy ingredients | 3.00 | 3.00 | .00 | 9 | 6.11 | 55.00 | 2 | 5.50 | 11.00 | -2.14 | **0.033** |
| Try a new recipe | 2.00 | 2.00 | .00 | 6 | 5.00 | 30.00 | 3 | 5.00 | 15.00 | -1.00 | 0.317 |
| Make recipes healthier | 2.00 | 2.00 | .00 | 15 | 9.07 | 136.00 | 2 | 8.50 | 17.00 | -3.13 | **0.002** |
| ***Knowledge*** |  |  |  |  |  |  |  |  |  |  |  |
| I know what to do in terms of healthy eating ^4^ | 4.00 | 5.00 | .00 | 15 | 9.27 | 139.00 | 2 | 7.00 | 14.00 | -3.13 | **0.002** |
| ***Self-efficacy*** *Are you able to … ^4^* |  |  |  |  |  |  |  |  |  |  |  |
| Cook healthy meals for your family? | 5.00 | 5.00 | .00 | 8 | 6.50 | 52.00 | 4 | 6.50 | 26.00 | -1.16 | 0.248 |
| Eat healthy with your family? | 5.00 | 5.00 | .00 | 7 | 5.71 | 40.00 | 3 | 5.00 | 15.00 | -1.39 | 0.166 |
| Cook with your child? | 5.00 | 5.00 | .00 | 15 | 9.13 | 137.00 | 2 | 8.00 | 16.00 | -3.12 | **0.002** |

^1^Based on negative ranks.
^2^ Scored from [1] ‘Never’ to [4] ‘Always’.
^3^ Sum scale not calculated due to unreliable scale.
^4^ Scored from [1] ‘No definitely not’ to [5] ‘Yes, definitely’.
